# The Gut Microbiota Reduces Colonization of the Mesenteric Lymph Nodes and IL-12-Independent IFN-γ Production During *Salmonella* Infection

**DOI:** 10.3389/fcimb.2015.00093

**Published:** 2015-12-22

**Authors:** María Fernández-Santoscoy, Ulf A. Wenzel, Ulf Yrlid, Susanna Cardell, Fredrik Bäckhed, Mary Jo Wick

**Affiliations:** ^1^Department of Microbiology and Immunology, Institute of Biomedicine, Sahlgrenska Academy, University of GothenburgGothenburg, Sweden; ^2^Sahlgrenska Center for Cardiovascular and Metabolic Research/Wallenberg Laboratory and the Department of Molecular and Clinical Medicine, Institute of Medicine, Sahlgrenska Academy, University of GothenburgGothenburg, Sweden

**Keywords:** *Salmonella*, microbiota, germ-free mice, infection, IFN-γ production

## Abstract

The intestinal commensal microbiota is essential for many host physiological processes, but its impact on infectious diseases is poorly understood. Here we investigate the influence of the gut microbiota during oral *Salmonella* infection. We report a higher bacterial burden in mesenteric lymph nodes (MLN) of intragastrically infected germ-free (GF) mice compared to conventionally-raised (CONV-R) animals, despite similar inflammatory phagocyte recruitment. *Salmonella* penetration into the lamina propria of the small intestine and splenic bacterial burden were not altered in the absence of the microbiota. Intragastrically infected GF mice also displayed a higher frequency of IFN-γ-producing NK, NKT, CD4^+^, and CD8^+^ T cells in the MLN despite IL-12 levels similar to infected CONV-R mice. However, infecting mice intraperitoneally abrogated the difference in MLN bacterial load and IFN-γ-producing cells observed in intragastrically-infected animals. Moreover, mice treated with antibiotics (ABX) and intragastrically infected with *Salmonella* had a greater bacterial burden and frequency of IFN-γ-producing cells in the MLN. In ABX mice the number of *Salmonella* correlated with the frequency of IFN-γ-producing lymphocytes in the MLN, while no such correlation was observed in the MLN of infected GF mice. Overall, the data show that the lack of the microbiota influences pathogen colonization of the MLN, and the increased IFN-γ in the MLN of infected GF mice is not only due to the absence of commensals at the time of infection but the lack of immune signals provided by the microbiota from birth.

## Introduction

Approximately 90% of the cells in the human body are microbes that reside in environmentally exposed surfaces (Cani and Delzenne, 2011). The most colonized body surface is the gut, which harbors around 100 trillion microorganisms constituted by at least 1000 bacterial species (Frank and Pace, 2008). The gut microbiota benefits the host in many ways including contributing to metabolic functions (Tremaroli and Bäckhed, 2012; Karlsson et al., 2013; Wichmann et al., 2013), stimulating the intestinal and systemic immune compartments (Talham et al., 1999; Mazmanian et al., 2005) and maintaining mucosal homeostasis (Atarashi et al., 2008; Ivanov et al., 2009; Maynard et al., 2012).

In addition to these commensal influences on homeostasis, they also play a protective role against invading pathogens. For example, competition for space, increasing competition for nutrients and induction of IgA are general ways that commensals help limit pathogen colonization (Srikanth and McCormick, 2008; Jarchum and Pamer, 2011; Haag et al., 2012; Buffie and Pamer, 2013). Ways that *Salmonella* subverts competition with commensals include producing energy more efficiently using products of reactive oxygen species released as a result of inflammation (Winter et al., 2010). Another example is the ability of *Salmonella* to acquire the essential metal zinc, despite zinc sequestration by calprotectin produced by neutrophils recruited during infection, through a high affinity zinc transporter (Liu et al., 2012). However, recolonizing germ-free mice (GF) with species-specific microbiota restored the intestinal immune compartment and reduced *Salmonella* fecal CFUs 4 days post infection (Chung et al., 2012).

Commensal-induced Th17 cells also contribute to pathogen defense by producing IL-17 and IL-22, which promote antibody class switching in B cells, induce anti-microbial peptides and contribute to neutrophil recruitment (Jarchum and Pamer, 2011; Littman and Pamer, 2011; Maynard et al., 2012). The microbiota also sends signals to immune cells in the intestinal compartment through pattern recognition receptors such as TLRs. For example, TLR9 engagement by commensal DNA promotes IL-17 and IFN-γ-producing T cells and reduces Treg frequency (Hall et al., 2008), which could facilitate immunity to pathogens. Intestinal dendritic cells also receive TLR-mediated signals from commensals that promote Th1 immunity (Benson et al., 2009) or induce IL-22 and IL-23 that could enhance mucosal defense (Kinnebrew et al., 2012). Moreover, evidence that the intestinal microbiota influences peripheral immune responses is mounting. For example, commensals influence the systemic immune response to viruses by inducing expression of inflammatory genes in phagocytes required for protective immunity (Abt et al., 2012; Ganal et al., 2012). Recognition of circulating, commensal-derived cell wall components can also enhance systemic innate immunity, killing of Gram positive pathogens, and induce monocyte emigration from the bone marrow (Clarke et al., 2010; Shi et al., 2011).

Despite these ways that commensals promote immunity to invading pathogens, they can also promote infection. For example, hatching of *Trichuris muris* eggs in the intestine requires contact with commensals (Hayes et al., 2010). Another parasite, *Heligmosomoides polygyrus*, can establish itself more successfully because of the elevated Treg frequency caused by commensal Lactobacillus (Reynolds et al., 2014). Viral infections can also be promoted by the microbiota. For instance, poliovirus relies on commensals for efficient replication while mouse mammary tumor virus binding to commensal LPS induces IL-10 production in splenocytes that promotes virus transmission (Kane et al., 2011; Kuss et al., 2011). Regarding bacterial pathogens, a study showed that the increased levels of sialic acid induced by the microbiota promote *Salmonella enterica* Serovar typhimurium (*S. typhimurium*) and *Clostridium difficile* expansion (Ng et al., 2013).

Pathogens can alter the steady-state composition of the microbiota and promote pathogenesis. For example, colonization by *Citrobacter rodentium* or *S. typhimurium* benefits from the modified proportion of commensal species caused by host-mediated inflammation (Lupp et al., 2007; Stecher et al., 2007). In addition, infection by *S. typhimurium* is favored by the effects in microbiota composition caused by IL-22 (Behnsen et al., 2014). Finally, IFN-γ production during *Toxoplasma gondii* infection eliminates Paneth cells, resulting in uncontrolled expansion of commensal *Enterobacteriaceae* and favoring *T. gondii* pathology (Raetz et al., 2013).

Studies thus far examining the interactions between *Salmonella* and intestinal commensals have primarily focused on the initial colonization stage, with little focus on the effects of the microbiota at later stages of the infection. The aim of this study was to elucidate the impact of the intestinal microbiota on the immune response to *Salmonella* 1 week post infection in GF mice.

## Materials and methods

### Mice

Germ-free (GF) C57BL/6J mice of both genders were kept in isolators at the Germ-free unit of the Laboratory for Experimental Biomedicine facility at the University of Gothenburg under a 12-h light cycle. Age- and gender-matched conventionally-raised (CONV-R) C57BL/6J mice were transferred to identical isolators at weaning. Both groups were fed an autoclaved low-fat polysaccharide-rich chow diet *ad libitum*. The mice used for the antibiotic experiments are wild-type C57BL/6J mice bred at the Experimental Biomedicine facility at the University of Gothenburg where they were provided with food and water *ad libitum*. The antibiotic treatment consisted of a cocktail containing bacitracin, neomycin and streptomycin (200 mg/Kg body weight of each antibiotic) administered intragastrically each morning for 3 days as previously described (Wichmann et al., 2013). These mice are called ABX mice. Control mice were given PBS instead of antibiotics. Animals were infected 24 h after the last antibiotic or PBS treatment. Mice were used between 8 and 12 weeks of age. All experiments were performed following protocols approved by the government animal ethics committee (permit 212-2013) and were performed using institutional animal care and use guidelines.

### Bacteria and animal infections

Mice were infected intragastrically with 3–5 × 10^7^ or via the intraperitoneal route (ip) with 1 × 10^7^ *S. enterica* serovar typhimurium χ4550 expressing OVA (Sundquist and Wick, 2009) (called *S. typhimurium*) grown overnight in LB media at 37°C with rotation. The bacterial concentration was determined spectrophotometrically by determining the OD_600_ and diluted to the desired concentration in sterile PBS. The actual bacterial dose was determined by plating serial dilutions of the bacterial suspension on LB agar plates. Animals infected intragastrically were first given 100 μl of 1% NaHCO_3_ intragastrically and 10 min later the bacterial dose was administered in 100 μl of PBS. Mice were sacrificed at the specified days post infection and the bacterial burden in the Peyer's patches (PP), small intestine lamina propria (siLP), mesenteric lymph nodes (MLN), and the spleen was determined by plating serial dilutions of cell suspensions on LB agar plates.

### Preparation of cell suspensions

To remove epithelial cells, the small intestine was incubated in HBSS (Gibco, Carlsbad, CA) containing 2 mM EDTA, 2% FCS (PAA Laboratories, Pasching, Austria), and 1 mM DTT (Sigma, St. Louis, MO). This treatment was repeated twice for 20 min at 37°C with gentle agitation. The tissue was washed once with HBSS to remove EDTA and then transferred to a digestion solution containing 10% FCS, 60 CDU/ml collagenase D (Roche), 60 kunitz units/ml DNase I (Roche, Basel, Switzerland), 0.28 CDU/ml Dispase (Roche), and 1 mM CaCl_2_ in HBSS for 20 min at 37°C with gentle agitation. The contents were transferred to gentleMACS tubes (Miltenyi Biotech, Bergisch Gladbach, Germany) and disrupted on a gentleMACS dissociator (Miltenyi Biotech). After filtration, single cell suspensions were obtained. MLN and PP were transferred to 5 ml of the digestion solution, incubated at 37°C for 45 min with agitation and the digested organs were pipetted into single cell suspensions. Spleens were incubated with 1 ml of digestion solution for 30 min at 37°C and then pressed through a nylon mesh to obtain single cells. Erythrocytes were lysed with 2 ml of a hypotonic solution of NH_4_Cl. All cell suspensions were washed once with HBSS and resuspended in sterile PBS. Cells were counted using a Sysmex KX-21N cell counter (Sysmex Corporation, Kobe, Japan).

### Antibodies

The following antibodies were used: CD11b-APC-Cy7 (M1/70), CD19-Biotin (6D5), TCRβ-Biotin (H57-597), NK1.1-Biotin (PK136), Ly6C-BV570 (HK1.4), Ly6G-PerCp-Cy5.5 (1A8), and TCRβ-Pacific Blue (H57-597) from Biolegend (San Diego, CA) CD8α-PE-Cy7 (53-6.7), CD4-Alexa 700 (RM4-5), and CD19-FITC (1D3) from BD Pharmingen (San Diego, CA); CD49b (DX5) from eBioscience (San Diego, CA) and MHC-II-Biotin (produced in house from hybridoma M5/114). The secondary Streptavidin-PE-CF594 from BD Horizon was used with biotin-conjugated primary antibodies. Antibody for detection of intracellular IFN-γ was IFN-γ-APC (XMG1.2) and the isotype control was Rat IgG1, κ-APC (RTK2071) (both from Biolegend). PE-labeled CD1d-tetramers loaded with α-galactosyl ceramide (αGalCer) (PBS57) were provided by the NIH Tetramer Facility and used to detect NKT cells.

### Flow cytometry

Non-viable cells were excluded using the *LIVE/DEAD* fixable *Aqua* dead cell stain kit (Life Technologies, Carlsbad, CA). Samples were then incubated with anti-FcγRII/III (2.4G2) purified as described previously (Yrlid and Wick, 2002) for 30 min at 4°C. Antibody cocktails were prepared in FACS buffer [HBSS containing 3% FCS, 10 mM Hepes (HyClone, Waltham, MA) and 5 mM EDTA (Sigma-Aldrich)]. Stainings were performed at 4°C for 20 min with the exception of αGalCer-CD1d tetramers, which was performed at room temperature for 30 min. FACS buffer was also used for all washing steps. For the detection of intracellular IFN-γ, cells were treated with Fixation/Permeabilization reagents (eBioscience) and stained intracellularly with anti-IFN-γ diluted in permeabilization buffer (eBioscience). Samples were acquired on a LSR-II flow cytometer (BD Biosciences) using DIVA software (BD Biosciences) and were analyzed using FlowJo software (Tree Star Inc., Palo Alto, CA).

### Quantitating IL-12p70 in whole MLN lysates

A saponin extract of MLN from *Salmonella*-infected mice at day 6 p.i. was made by collecting MLN in 0.05% PBS-Tween containing Pefabloc SC (Roche), soybean trypsin inhibitor, bovine serum albumin and EDTA (all from Sigma). MLN were weighed, Saponin (Sigma) was added to a final concentration of 2% (w/v) in PBS and samples were incubated overnight at 4°C. The samples were then centrifuged at 13,000 × g for 10 min and supernatants were collected and stored at −20°C until analysis. IL-12p70 was quantitated using the Bio-Plex Pro Mouse Cytokine IL-12p70 set from Bio-Rad (Hercules, CA) according to manufacturer's recommendation. The data was acquired in a MAGPIX instrument from Luminex (Austin, TX) and analyzed with the xPONENT software by Luminex.

### Statistics

Statistical analysis was performed using GraphPad Prism (GraphPad Software, La Jolla, CA). Each group was compared against the control group using the two-tailed nonparametric Mann-Whitney-U test. For correlations (**Figure 4**), the two variables were compared using Spearman correlation. *p* < 0.05 were considered significant. ^*^p < 0.05; ^**^p < 0.01; ^***^p < 0.001; ^****^p < 0.0001.

## Results

### The MLN of GF and ABX mice have a higher *Salmonella* burden compared to controls

**Figure 1:**
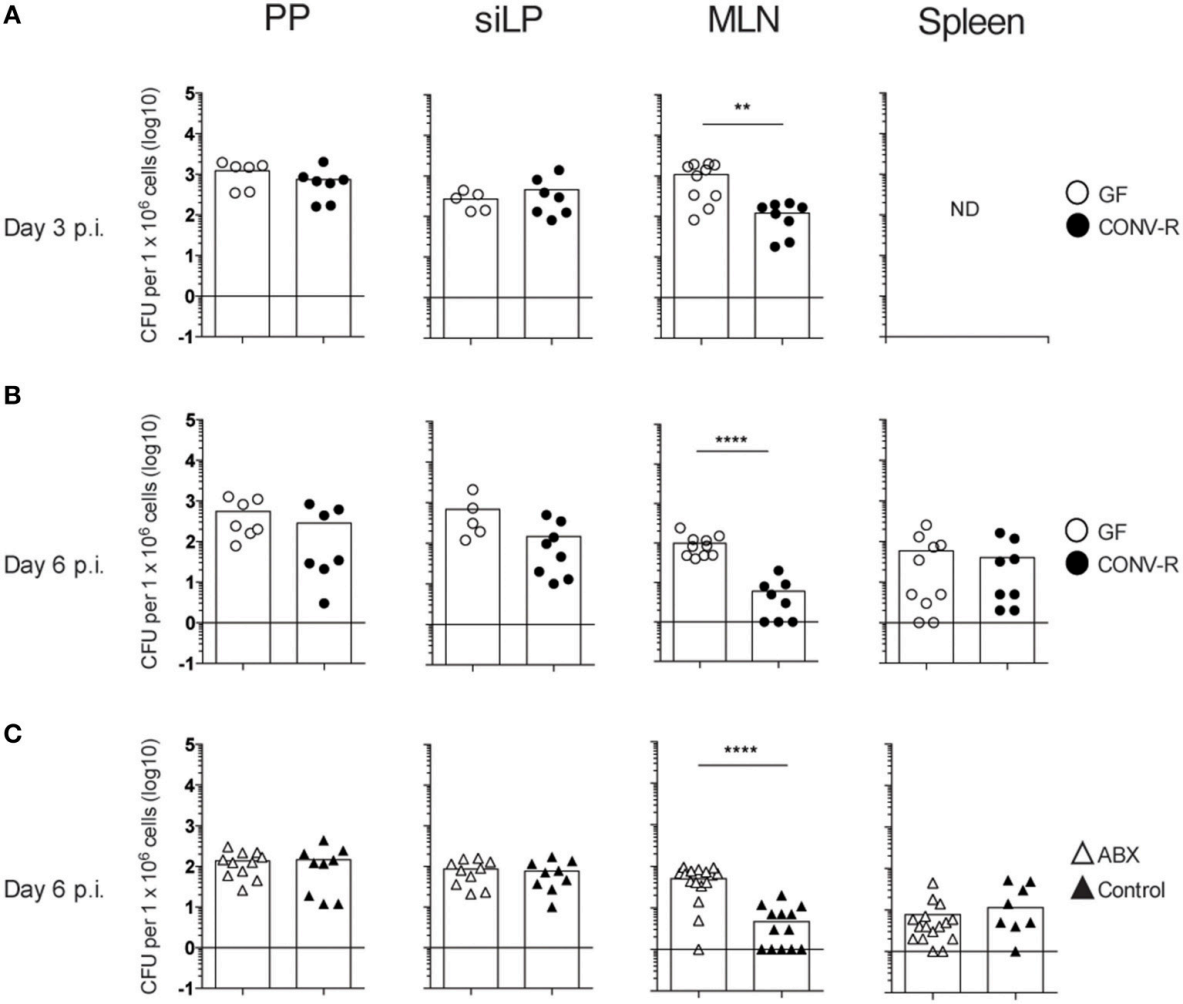
**The MLN of ***Salmonella***-infected GF and ABX-treated mice have a higher bacterial burden compared to controls**. Mice were intragastrically infected with 3–5 × 10^7^ *S. typhimurium* and at day 3 **(A)** and day 6 **(B,C)** p.i., the bacterial load in the organs was assessed. **(A,B)** CFUs in PP, siLP, and MLN from GF and CONV-R mice at day 3 **(A)** or day 6 **(B)** p.i. is shown. In **(A)**, CFUs in the spleen at day 3 pi were not determined (ND). **(C)** CFUs in PP, siLP, MLN, and spleen from ABX and control mice at day 6 p.i. are shown. Each symbol represents an individual mouse and bars are the mean. Empty circles are GF, filled circles are CONV-R, empty triangles are ABX and filled triangles are PBS-treated controls. Data in **(A)** are pooled from two experiments with 2–4 mice per group in each experiment; **(B)** two experiments with 2–6 mice per group in each experiment; and **(C)** three experiments with 3–7 mice per group in each experiment. ^**^p < 0.01; ^****^p < 0.0001.

To assess the role of the microbiota in the host response to *Salmonella*, the bacterial burden in intestinal tissues and the spleen was determined in infected GF and CONV-R mice. GF and CONV-R mice were intragastrically infected with *S. typhimurium* and the bacterial load in PP, siLP, MLN, and spleen were determined at days 3 and 6 post infection (p.i.). A higher bacterial burden was observed in MLN of GF mice compared to CONV-R mice at both time points after infection (Figures 1A,B). In contrast, there were no significant differences in CFUs in PP and siLP of GF and CONV-R mice at days 3 or 6 p.i. or in spleen at day 6 p.i. (Figures 1A,B).

Altered numbers of inflammatory monocytes and neutrophils did not seem to account for the increased bacterial burden in the MLN of GF mice. That is, infected MLN of GF and CONV-R mice exhibited similar numbers of Ly6G^hi^ neutrophils, Ly6C^int^Ly6G^−^ resident monocytes and Ly6C^high^Ly6G^−^ inflammatory monocytes at 3, 6 and 13 days p.i. (Figure S1). We next addressed whether the higher CFUs in the MLN of GF mice was due to diminished microbiota *per se* or other alterations in the host response of GF mice due to the absence of the microbiota from birth, which influences the intestinal immune system. Thus, adult CONV-R mice were treated with antibiotics as previously described (Wichmann et al., 2013) to reduce the gut microbiota and were intragastrically infected with *Salmonella*. Similar to the results with GF mice, higher CFUs were found in MLN, but not in PP, siLP, or spleen of ABX mice compared to controls at day 6 p.i. (Figure 1C). Thus, both GF and ABX mice display higher *Salmonella* loads in MLN compared to their respective controls.

### The MLN of *Salmonella*-infected GF and ABX mice display a higher frequency of IFN-γ-producing cells compared to their controls despite similar IL-12p70

**Figure 2:**
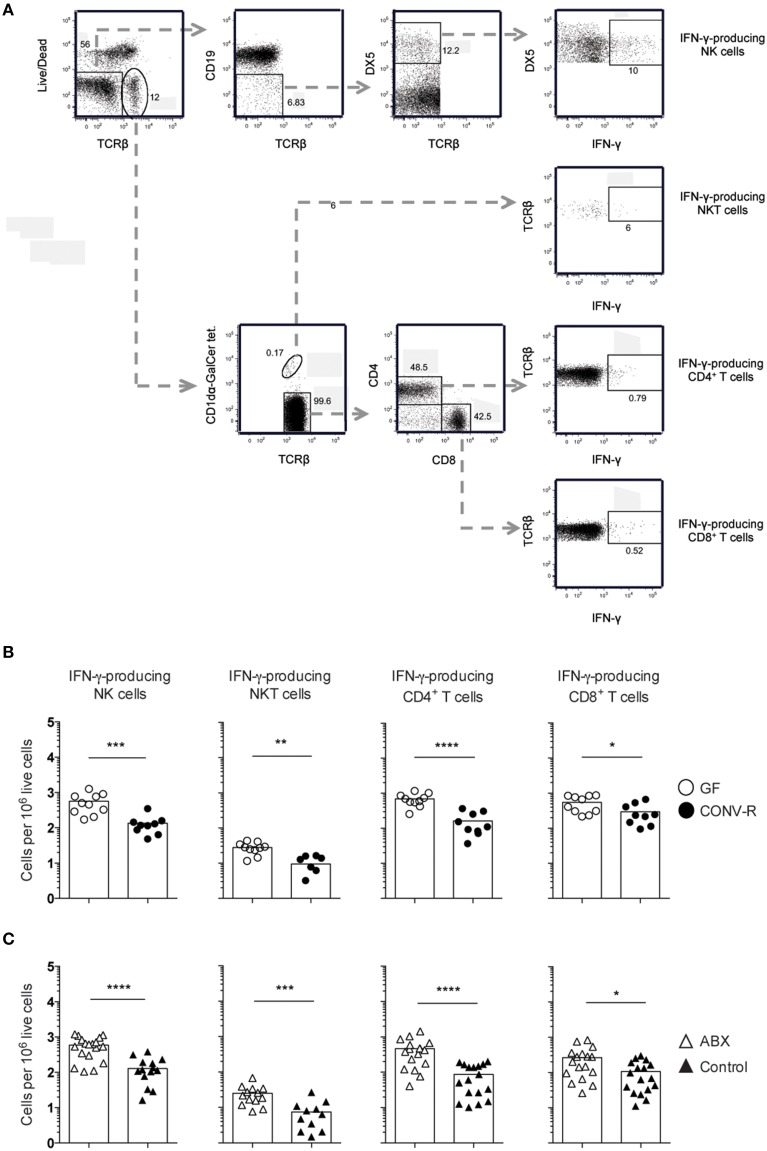
**Increased frequency of IFN-γ-producing lymphocytes in the MLN of GF and ABX mice intragastrically infected with ***Salmonella*****. Mice were intragastrically infected with 3–5 × 10^7^ *S. typhimurium* and at day 6 p.i. the MLN were dissected and single cell suspensions were stained with Live-dead Aqua, CD1dα-GalCer tetramers and anti- TCRβ, CD19, DX5, CD4, CD8α, and IFN-γ. **(A)** Gating strategy used to identify viable IFN-γ^+^ cells among (top to bottom) NK cells, NKT cells, CD4^+^ T cells, and CD8^+^ T cells in the MLN of an ABX mouse is shown. **(B,C)** The frequency of IFN-γ-producing cells in the indicated populations from GF and CONV-R mice **(B)** and from ABX and control mice **(C)** is shown. Symbols represent individual mice and bars are the mean. Empty circles are GF, filled circles are CONV-R, empty triangles are ABX and filled triangles are PBS-treated controls. Data in **(B)** are pooled from two experiments with 4–6 mice per group in each experiment except for NKT cells in CONV-R mice where 3–4 mice were used per experiment. Data in **(C)** are pooled from three experiments with 4–7 mice per group in each experiment except for NKT cells in control mice where 3–4 mice were used per experiment. ^*^p < 0.05; ^**^p < 0.01; ^***^p < 0.001; ^****^p < 0.0001.

**Figure 3:**
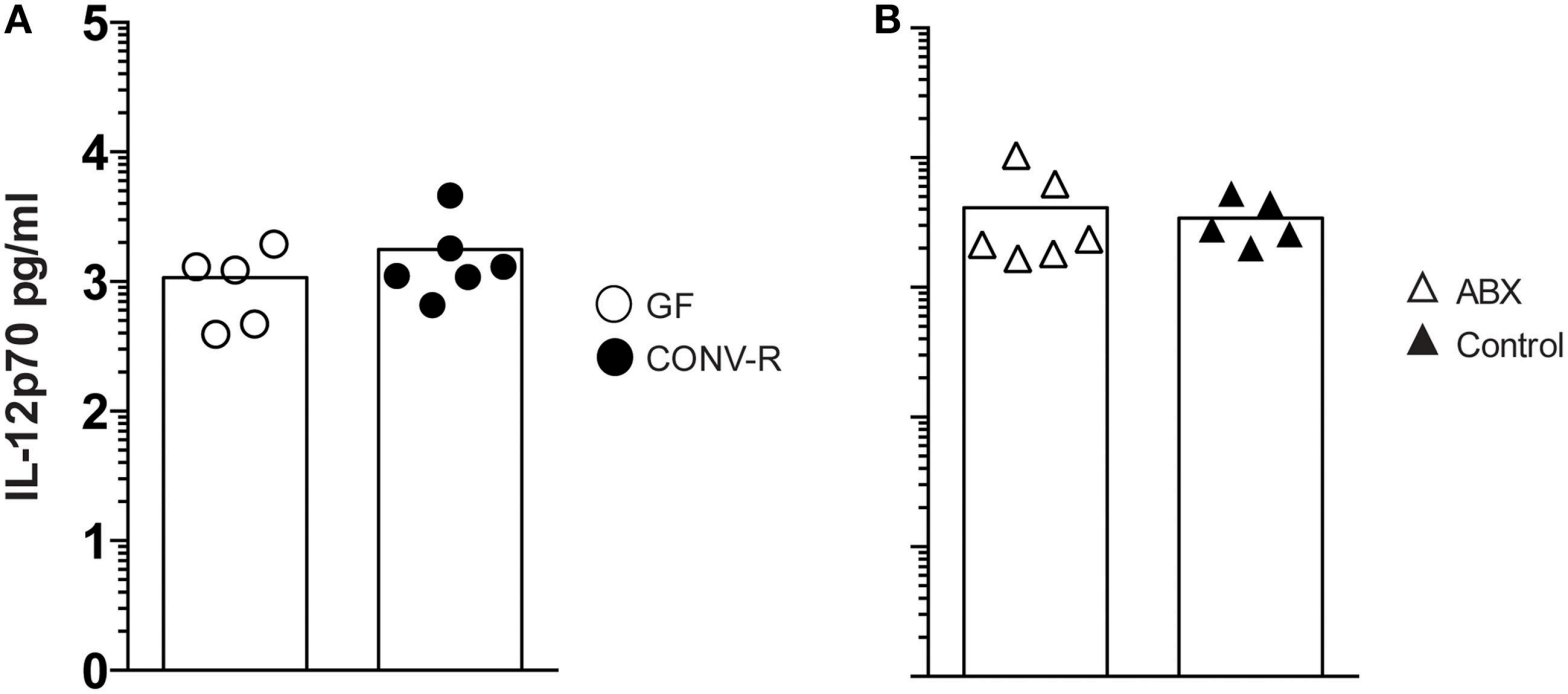
**The MLN of ***Salmonella***-infected GF and ABX mice have similar IL-12 levels as the MLN of their respective control**. Mice were intragastrically infected with 3–5 × 10^7^ *S. typhimurium* and at day 6 p.i. IL-12p70 in whole MLN lysates was quantitated. Data from infected GF and CONV-R mice **(A)** and from infected ABX and controls **(B)** normalized to 1 g tissue are shown. Symbols represent individual mice and bars are the mean. Empty circles are GF, filled circles are CONV-R, empty triangles are ABX and filled triangles are controls. Data show one representative of two independent experiments that examined 3–6 mice per group in each experiment.

The frequency of IFN-γ-producing NK and NKT cells, as well as CD4^+^ and CD8^+^ T cells, was measured in the MLN of *Salmonella*-infected GF, CONV-R, ABX, and control mice at day 6 p.i. The MLN of infected GF mice had a higher frequency of IFN-γ-producing cells among all four populations compared to CONV-R mice although the contribution of NKT cells to IFN-γ production was very small at this time point (Figures 2A,B). Similar results were found in infected ABX mice compared to controls (Figure 2C). The increase in the frequency of IFN-γ-producing CD4^+^ and CD8^+^ T cells in the MLN of infected GF mice occurred despite a lower number of CD4^+^ and CD8^+^ T cells in this tissue compared to CONV-R mice at day 6 p.i. (Figure S2A). In contrast, no differences in the number of CD4^+^ and CD8^+^ T cells in the MLN of ABX compared to control mice was apparent (Figure S2B). Surprisingly, intragastrically infected GF and ABX mice had similar concentrations of IL-12p70 in MLN compared to their respective controls 6 days p.i. (Figure 3). Thus, *Salmonella*-infected GF and ABX mice have a higher frequency of IFN-γ-producing NK, NKT, CD4^+^, and CD8^+^ T cells compared to their respective infected controls. Moreover, differences in IL-12 production in the MLN of GF and ABX mice do not seem to account for the higher levels of IFN-γ compared to their controls.

**Figure 4:**
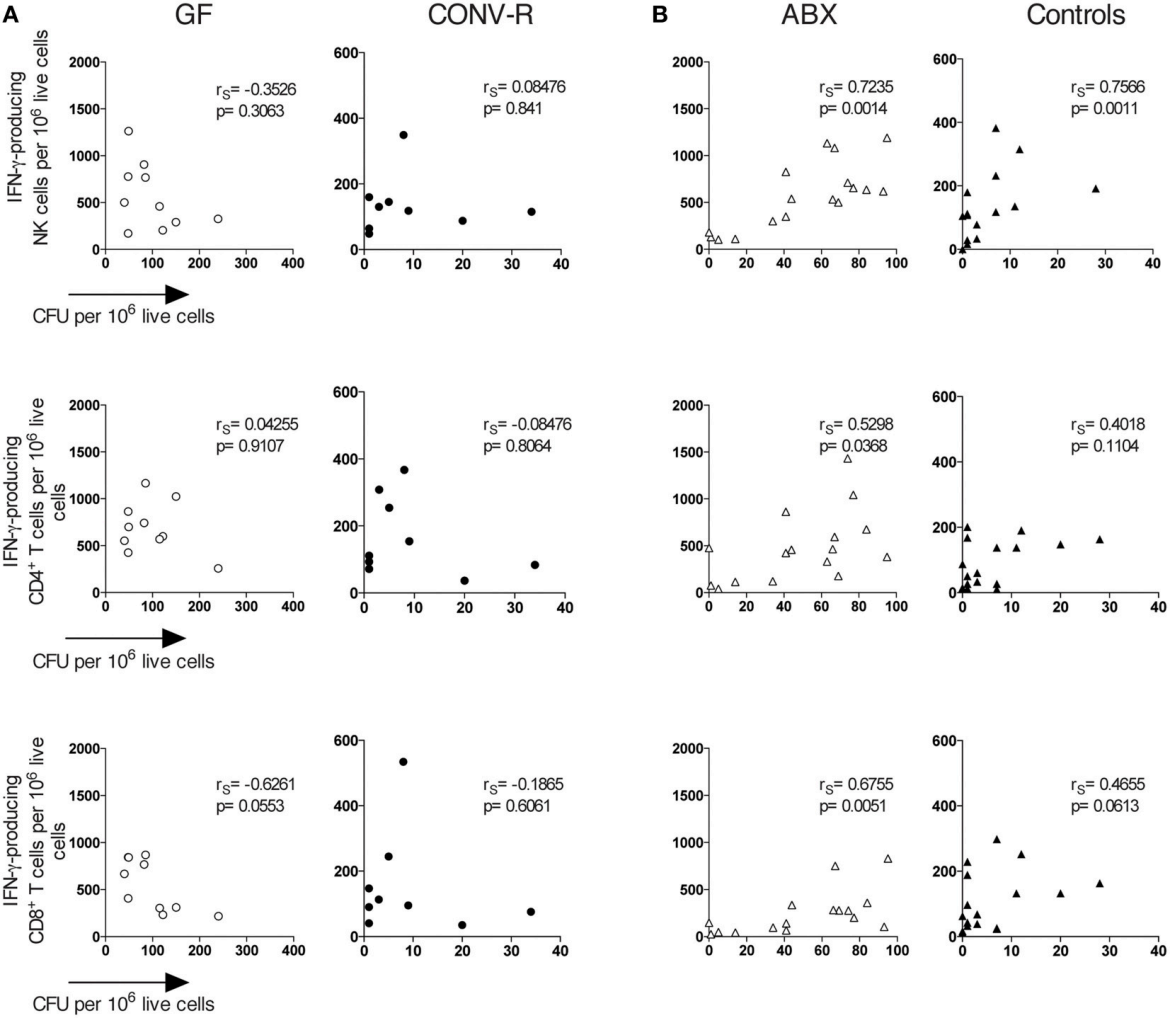
**A correlation exists between CFUs and the frequency of IFN-γ-producing lymphocytes in the MLN of ***Salmonella***-infected ABX but not GF mice**. Mice were infected intragastrically with *S. typhimurium* and day 6 p.i. the MLN were dissected and single cell suspensions were prepared. Bacterial loads were assessed as CFUs and FACS analysis was performed to determine the frequency of IFN-γ-producing NK, CD4^+^, and CD8^+^ T cells in GF and CONV-R mice **(A)** and in ABX and control mice **(B)**. Symbols represent individual mice. Empty circles are GF, filled circles are CONV-R, empty triangles are ABX and filled triangles are controls. Data are pooled from 2 to 3 experiments with 4–7 mice per group in each experiment. *r*_*S*_ values indicate Spearman correlation coefficients.

The number of IFN-γ-producing cells in the MLN of infected GF and ABX mice was rather heterogeneous (Figures 2B,C, open symbols), yet as a group the animals had significantly more IFN-γ-producing cells compared to their respective controls (Figures 2B,C). We thus wished to directly determine the relationship between bacterial burden and the frequency of IFN-γ-producing cells in the same individual mouse. We thus analyzed the correlation between bacterial burden and frequency of IFN-γ-producing NK cells, CD4^+^ T cells, and CD8α^+^ T cells in individual, intragastrically-infected GF, CONV-R, ABX, and control mice using Spearman correlation analysis. This showed no correlation between the number of CFUs and the frequency of IFN-γ-producing cells in the MLN of infected GF mice (Figure 4A). However, a correlation exists between CFUs and frequency of IFN-γ-producing cells in the MLN of infected ABX mice analyzed as individuals (Figure 4B). Thus, *Salmonella*-infected ABX-treated and GF mice, analyzed as groups, have a higher frequency of IFN-γ-producing lymphocytes in the MLN relative to their respective controls. However, when individual animals are analyzed, increased CFUs and IFN-γ-producing cells in MLN correlate in infected ABX but not in infected GF mice.

### Intraperitoneal infection results in CFUs and IFN-γ-producing cells in the MLN of GF and ABX mice that are similar to their controls

**Figure 5:**
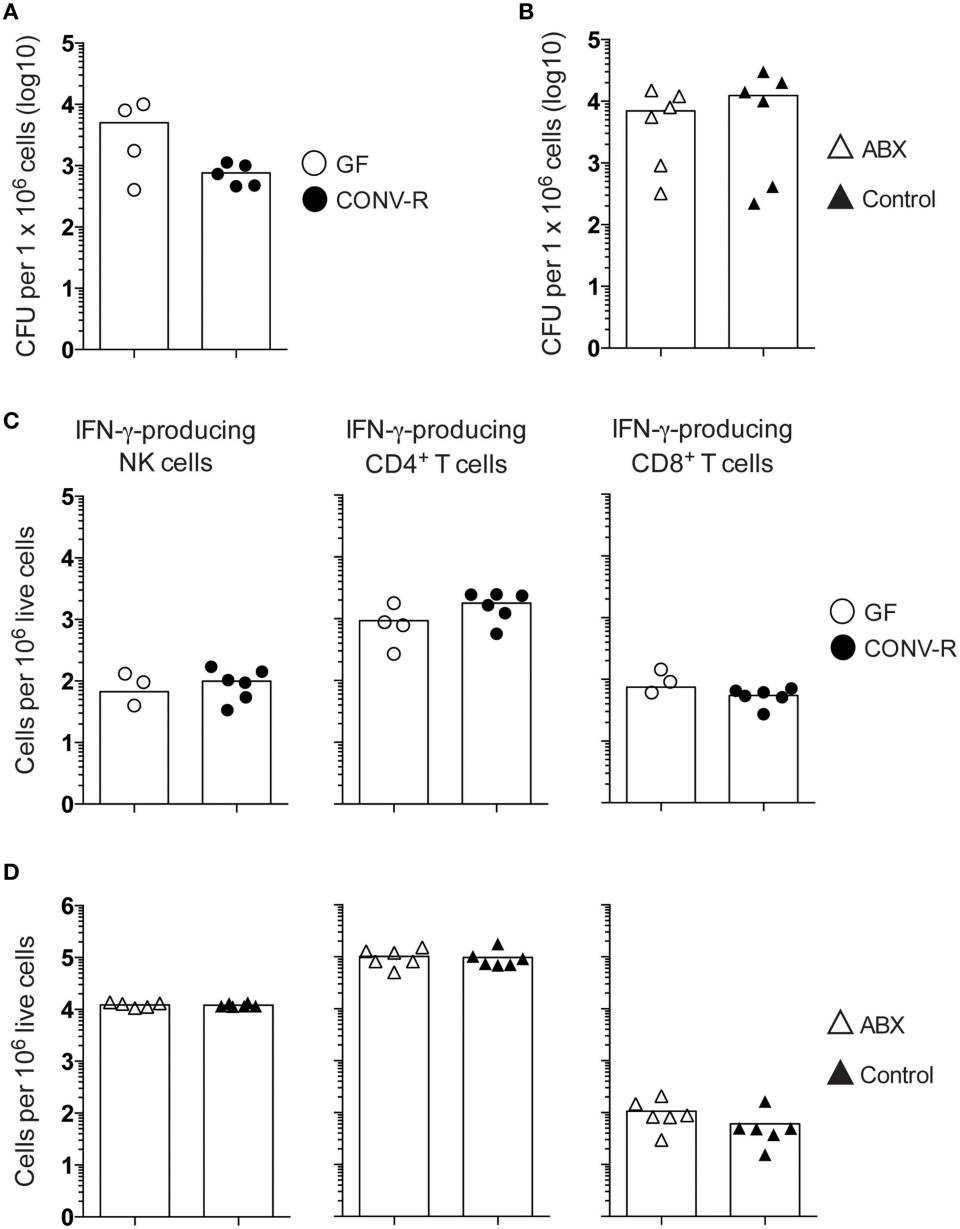
**The MLN of IP-infected GF and ABX mice have a similar frequency of IFN-γ-producing NK cells, CD4^**+**^ T cells, and CD8^**+**^ T cells compared to their controls**. Mice were IP infected with 1 × 10^7^ *S. typhimurium* and at day 6 p.i. the MLN were dissected and single cell suspensions were prepared. The bacterial loads were assessed as CFUs from **(A)** GF and CONV-R mice and **(B)** ABX and control mice. **(C,D)** FACS analysis was performed to determine the frequency of IFN-γ-producing NK cells, CD4^+^ T cells, and CD8^+^ T cells from **(C)** GF and CONV-R mice and from **(D)** ABX and control mice. The gating strategy is as in Figure 2A. Symbols represent individual mice and bars are the mean. Empty circles are GF, filled circles are CONV-R, empty triangles are ABX and filled triangles are controls. Data in **(A,C)** show one representative experiment out of two with 2–6 mice per group in each experiment and in **(B,D)** show one representative experiment out of three with 4–6 mice per group in each experiment.

We next infected GF and ABX mice by the IP route. In contrast with the results obtained after oral infection, IP-infected GF and CONV-R mice, as well as IP-infected ABX and control mice, displayed similar CFUs in the MLN (Figures 5A,B). Moreover, no difference in frequency of IFN-γ-producing NK cells, CD4^+^ T cells or CD8^+^ T cells was found in the MLN of infected GF mice compared to CONV-R mice at day 6 p.i. (Figure 5C). Similarly, the MLN of IP-infected ABX and control mice displayed comparable frequencies of IFN-γ-producing NK cells, CD4^+^ T cells and CD8α^+^ T cells at the same time point (Figure 5D). Thus, in an IP infection where the effects of commensals in initial bacterial penetration across the intestinal barrier is circumvented, GF mice, ABX mice and their respective controls show equal bacterial loads and frequency of IFN-γ-producing cells in MLN.

## Discussion

The microbiota has a significant influence on the generation of the intestinal immune compartment and mucosal homeostasis yet its impact on infectious diseases is poorly understood. Using an oral *Salmonella* infection model, we observed that bacterial loads in PP and siLP were similar in infected GF and CONV-R mice. This suggests that initial bacterial penetration and seeding of these tissues is not affected by the absence of intestinal microbiota. Furthermore, *Salmonella* CFUs in the spleen of GF and CONV-R mice was similar at day 6 and 13 (not shown) p.i., suggesting that the capacity to seed systemic tissue is not altered in mice reared in the absence of intestinal microbiota. In contrast, a higher bacterial burden was observed in the MLN of GF compared to CONV-R mice at days 3 and 6 p.i. Similar recruitment of neutrophils and inflammatory monocytes in the MLN of infected GF and CONV-R animals supports that aberrant recruitment of phagocytes important in controlling initial *Salmonella* replication (Conlan, 1997; Yang et al., 2002; Cheminay et al., 2004; Rydström and Wick, 2007, 2009) does not account for increased CFUs in the MLN of GF mice early during infection. An additional possibility is that GF mice have a compromised production of anti-microbial peptides that contributes to increased *Salmonella* CFUs in the MLN of these animals. However, cells within the intestinal epithelium rather than immune cells produce these peptides (Cunliffe and Mahida, 2004; Bevins and Salzman, 2011) and therefore differences in CFUs in the siLP of infected GF and CONV-R mice would also be predicted. This, however, was not observed.

Several general mechanisms, including competition for space and nutrients as well as induction of IgA, are ways commensals help limit pathogen colonization (Kamada et al., 2013). These general mechanisms could account for the higher *Salmonella* CFUs in the MLN of infected GF mice. To address this using a different means to reduce intestinal commensals, we infected mice pretreated with antibiotics intragastrically (Wichmann et al., 2013). In this way we could reduce the microbiota of adult mice reared in a conventional environment and whose immune system was exposed to the natural influence of the microbiota from birth. Similar to GF mice, and consistent with previously published data using noninvasive *Salmonella* (Diehl et al., 2013), ABX mice also displayed higher *Salmonella* CFUs in MLN compared to untreated controls. Our data are also supported by recent reports which showed that altering the intestinal microbiota with, for example, antibiotic treatment resulted in increased penetration of colonic commensals, via goblet cell-associated antigen passages, to MLN (Knoop et al., 2015a,b). Thus, short-term oral antibiotic treatment increases susceptibility to intestinal bacteria and promotes penetration to the MLN.

To further address mechanisms underlying enhanced CFUs in the MLN of infected GF and ABX mice, we turned to effector mechanisms required to eliminate *Salmonella*. IFN-γ is essential in host defense against *Salmonella* (Coburn et al., 2007) and IFN-γ-producing cells, particularly CD4^+^ TCRαβ T cells (Nauciel and Espinasse-Maes, 1992; Muotiala and Mäkelä, 1993; Hess et al., 1996), are important effectors in conferring host protection to *Salmonella* (Moon and McSorley, 2009). Consistent with the reduced T cells GF mice have in the intestinal immune compartment in steady state (Cash and Hooper, 2005; Round and Mazmanian, 2009), we observed reduced total (TCRβ^+^) as well as reduced CD4^+^ and CD8^+^ T cells in the MLN of GF mice infected 6 days earlier with *Salmonella*. Despite this, intragastrically infected GF and ABX mice had a higher frequency of IFN-γ-producing cells, particularly CD4^+^ and CD8^+^ T cells as well as NK cells, in the MLN compared to their respective controls. For GF mice this appeared to not simply be a consequence of the high bacterial numbers in the tissue since a correlation between CFUs and IFN-γ-producing cells was not apparent. However, a positive correlation was apparent for infected ABX mice. This suggests that GF mice have an increased IFN-γ-production that is not derived only from the absence of commensals at the time of the infection but by the lack of immune signals provided by the microbiota from birth. Overall the data of Knoop et al., Diehl et al., and our data support that disrupting intestinal microbial sensing by antibiotic treatment, raising mice under GF conditions or ablating signaling from commensals mediated by MyD88 seem to promote intestinal bacterial translocation to MLN with induction of inflammation including the production of IFN-γ (Diehl et al., 2013; Knoop et al., 2015a,b). The relative contribution of bacteria transported inside phagocytes and/or extracellular bacteria traveling in lymph to the increased bacterial load in MLN remains to be elucidated. Moreover, our data show that the influence of the microbiota on IFN-γ production in *Salmonella*-infected ABX or GF mice is independent of IL-12p70 levels in the MLN.

To further investigate the role of the microbiota in inducing IFN-γ production in the MLN, we infected mice IP. This bypasses the influence of the intestinal microbiota and penetration across the intestinal barrier in the initial phase of *Salmonella* infection. We found no difference in CFUs or the frequency of IFN-γ-producing cells in the MLN of IP-infected GF and ABX mice relative to their controls. This supports that the intestinal microbiota protects the host against oral *Salmonella* infection through several general mechanisms such as competition for space and/or nutrients. Furthermore, it also supports the role of intestinal commensals in limiting pathogen translocation to the MLN and induction of inflammation, including IFN-γ-producing cells, in the MLN (Knoop et al., 2015a,b).

Overall, this study shows that mice lacking or having reduced intestinal microbiota and intragastrically infected with *Salmonella* have higher CFUs in the MLN and an increased frequency of IFN-γ-producing cells. It provides evidence of an influence of the intestinal microbiota in limiting *Salmonella* translocation from the intestinal lumen and induction of inflammation. These results set the foundation for future investigation of the cross-talk between the intestinal microbiota and IFN-γ-producing cells and how this influences the host response to infection with oral pathogens.

## Author contributions

MF-S contributed to the design of the study, performed experiments, analyzed data, contributed to interpretation of the data and wrote drafts of the manuscript. UAW contributed to the design of the study and performed experiments. UY, FB, and MJW contributed to the conception and design of the study, interpreted data and contributed to writing of the manuscript. SC contributed to interpretation of the data and contributed unique reagents. All authors approved the final version of the manuscript.

### Conflict of interest statement

The authors declare that the research was conducted in the absence of any commercial or financial relationships that could be construed as a potential conflict of interest.
