# Supplementary material for: The Gut Microbiota Reduces Colonization of the Mesenteric Lymph Nodes and IL-12-Independent IFN-γ Production During Salmonella Infection
Source: Front Cell Infect Microbiol. 2015 Dec 22;5:93. doi: 10.3389/fcimb.2015.00093 (PMC4687475; doi:10.3389/fcimb.2015.00093)
Supplement: Supplementary file 1 [file DataSheet1.docx]

Supplementary Material

**The normal gut microbiota reduces colonization of the mesenteric lymph nodes and IL-12-independent IFN-γ production during *Salmonella* infection**

María Fernández-Santoscoy^1^, Ulf Alexander Wenzel^1^, Ulf Yrlid^1^, Susanna Cardell^1^, Fredrik Bäckhed^2^ and Mary Jo Wick^1*^

^1^Department of Microbiology and Immunology, Institute of Biomedicine, Sahlgrenska Academy, University of Gothenburg, Sweden; ^2^Sahlgrenska Center for Cardiovascular and Metabolic Research/Wallenberg Laboratory and the Department of Molecular and Clinical Medicine, Institute of Medicine, Sahlgrenska Academy, University of Gothenburg, Sweden

***Address correspondence to:**

Mary Jo Wick

Dept. Microbiology and Immunology

University of Gothenburg

Box 435

405 30 Gothenburg, Sweden

+46 31 786 6325

mary-jo.wick@immuno.gu.se

**Supplementary Figures**

**Figure S1. Recruitment of neutrophils and monocytes is not altered in the MLN of infected GF mice.** Mice were intragastrically infected with *S. typhimurium* and 3, 6 and 13 days p.i. the MLN were dissected and single cell suspensions were prepared. Cells were stained with Live-dead Aqua, an exclusion cocktail containing anti- CD19 and NK1.1 as well as anti-TCRβ, anti-CD11b, anti-Ly6C and anti-Ly6G. (A) Gating strategy showing viable cells that were exclusion cocktail negative but positive for CD11b (left panel) that were further gated for Ly6C and Ly6G (right panel). Neutrophils were defined as Ly6C^+^Ly6G^+^, inflammatory monocytes as Ly6C^+^Ly6G^-^ and resident monocytes as Ly6C^int^ Ly6G^-^ as shown. The sample is from a GF mouse at day 6 p.i.. (B) Neutrophil and (C) monocyte numbers at day 0, 3, 6 and 13 p.i. are shown. Data are the mean value ± SD of 2-3 experiments with 3-4 mice per group in each experiment, except for day 13 where 1 experiment with 3 mice per group is shown.

**Figure S2. The MLN of *Salmonella*-infected GF mice contain a reduced number of TCRβ^+^, CD4^+^ and CD8α^+^ T cells.** Mice were intragastrically infected with 3-5 x 10^7^ *S. typhimurium* and day 6 p.i. MLN were dissected and single cell suspensions were stained and gated as in Fig. 2A. (A) shows data from infected GF and CONV-R mice and (B) from infected ABX and controls. Symbols represent individual mice and bars are the mean. Empty circles are GF, filled circles are CONV-R, empty triangles are ABX and filled triangles are controls. Data in (A) show one representative out of two with 4-6 mice per group in each experiment and in (B) show one representative experiment out of three with 4-7 mice per group in each experiment.
