# Supplementary figures and images for: The Gut Microbiota Reduces Colonization of the Mesenteric Lymph Nodes and IL-12-Independent IFN-γ Production During Salmonella Infection
Source: Front Cell Infect Microbiol. 2015 Dec 22;5:93. doi: 10.3389/fcimb.2015.00093 (PMC4687475; doi:10.3389/fcimb.2015.00093)

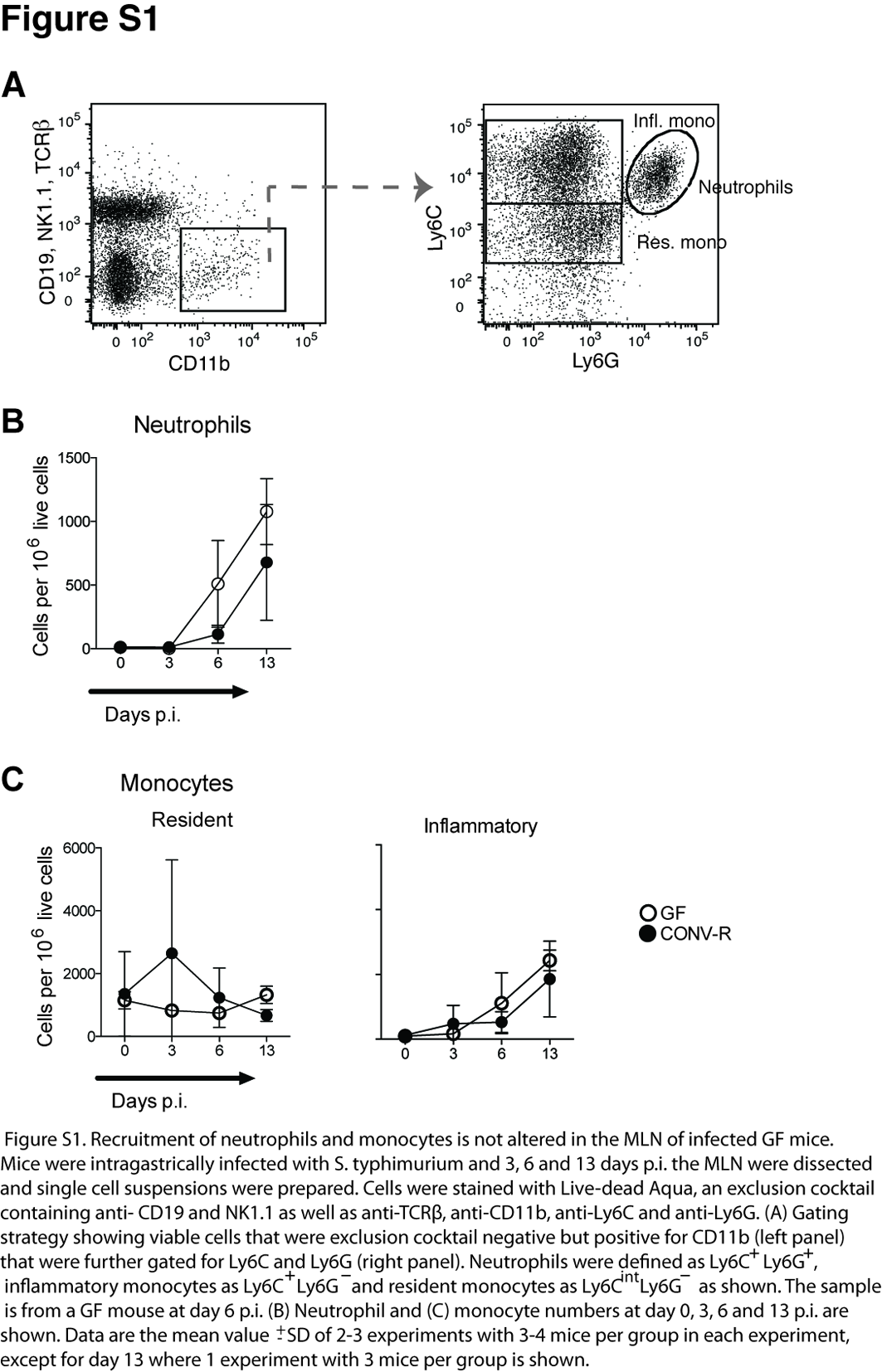

Supplement: Supplementary file 2 [file Image1.TIF]

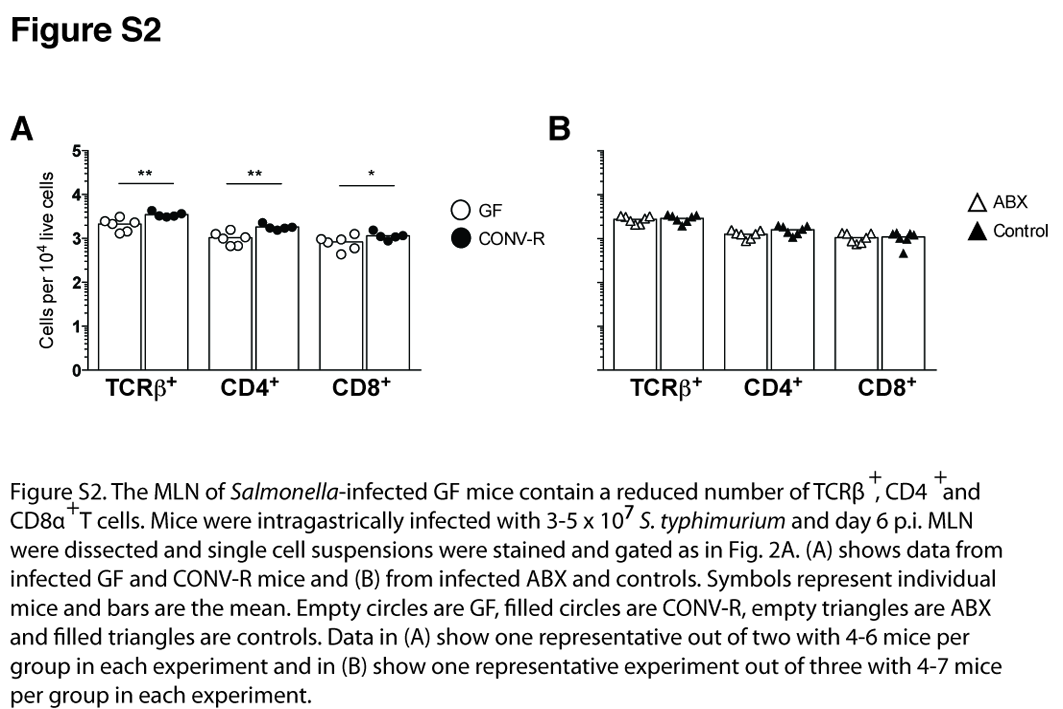

Supplement: Supplementary file 3 [file Image2.tiff]
